# Copy number variability of expression plasmids determined by cell sorting and Droplet Digital PCR

**DOI:** 10.1186/s12934-016-0610-8

**Published:** 2016-12-19

**Authors:** Michael Jahn, Carsten Vorpahl, Thomas Hübschmann, Hauke Harms, Susann Müller

**Affiliations:** 1 Helmholtz-Centre for Environmental Research-UFZ, Permoserstraße 15, 04318 Leipzig, Germany; 2 School of Biotechnology, Science for Life Laboratory, KTH-Royal Institute of Technology, Stockholm, Sweden

**Keywords:** *Escherichia coli*, SEVA, Replication system, Origin of replication, Plasmid copy number, EGFP, Population heterogeneity, Variability, Cell sorting, Sub-population

## Abstract

**Background:**

Plasmids are widely used for molecular cloning or production of proteins in laboratory and industrial settings. Constant modification has brought forth countless plasmid vectors whose characteristics in terms of average plasmid copy number (PCN) and stability are rarely known. The crucial factor determining the PCN is the replication system; most replication systems in use today belong to a small number of different classes and are available through repositories like the Standard European Vector Architecture (SEVA).

**Results:**

In this study, the PCN was determined in a set of seven SEVA-based expression plasmids only differing in the replication system. The average PCN for all constructs was determined by Droplet Digital PCR and ranged between 2 and 40 per chromosome in the host organism *Escherichia coli*. Furthermore, a plasmid-encoded EGFP reporter protein served as a means to assess variability in reporter gene expression on the single cell level. Only cells with one type of plasmid (RSF1010 replication system) showed a high degree of heterogeneity with a clear bimodal distribution of EGFP intensity while the others showed a normal distribution. The heterogeneous RSF1010-carrying cell population and one normally distributed population (ColE1 replication system) were further analyzed by sorting cells of sub-populations selected according to EGFP intensity. For both plasmids, low and highly fluorescent sub-populations showed a remarkable difference in PCN, ranging from 9.2 to 123.4 for ColE1 and from 0.5 to 11.8 for RSF1010, respectively.

**Conclusions:**

The average PCN determined here for a set of standardized plasmids was generally at the lower end of previously reported ranges and not related to the degree of heterogeneity. Further characterization of a heterogeneous and a homogeneous population demonstrated considerable differences in the PCN of sub-populations. We therefore present direct molecular evidence that the average PCN does not represent the true number of plasmid molecules in individual cells.

**Electronic supplementary material:**

The online version of this article (doi:10.1186/s12934-016-0610-8) contains supplementary material, which is available to authorized users.

## Background

Plasmids have been used in biotechnology for decades as they are easy to manipulate and transfer to host cells. Plasmids replicate autonomously from the bacterial chromosome and are usually present in more than one copy per cell, leading to higher recombinant gene dosage. However, the design and cloning of plasmid vectors in many laboratories world-wide did not follow any systematic rules [1]. The result is an overwhelming number of plasmid parts which are often poorly characterized. Those parts include antibiotic resistance cassettes, replication and induction systems, and a great number of ‘cargo’ genes. The essential part of a plasmid primarily determining its copy number (PCN) is the replication system, in most cases composed of a *vegetative* origin of replication (*oriV*), and a gene encoding the replication initiation protein (*rep*) [2].

For biotechnological applications it is highly desirable to know the range of copy numbers that can be expected from a particular vector, as the gene dosage can be crucial for efficient protein production [3]. A low mean PCN furthermore promotes failure of plasmid distribution to daughter cells [4], while a higher mean PCN is supposed to ensure that every daughter obtains plasmid molecules [5]. It must be noted that this behavior is different in low-copy plasmids that carry active partitioning systems to ensure faithful distribution of one or few plasmid copies from mother to daughter cells. This reduces heterogeneity, however, no such system was included in this study. An example of heterogeneity caused by loss of a very low-copy plasmid was the bimodal distribution of EGFP fluorescence in *Pseudomonas putida* [6, 7]. As plasmids follow a discrete distribution, a low mean PCN may also lead to higher cell to cell heterogeneity regarding PCN and gene expression, as e.g. shown by Kittleson and colleagues using a library of 20 *rep* mutants [8].

However, only sparse information regarding PCN and expression heterogeneity is available for the wealth of different replication systems used in laboratories world-wide. Nevertheless, there is a demand for standardized genetic parts with predictable function [9], and recently, efforts were undertaken to create platforms for systematic creation, annotation, and combination of such parts. Examples are e.g. the biobricks standard accompanied by its Registry of Standard Biological Parts [10] or the Standard European Vector Architecture (SEVA) for systematic assembly of plasmids [1, 11].

In this study, we chose the SEVA standard as underlying architecture for plasmid design, as this repository provides a coherent modular plasmid structure and a wealth of replication systems that can be used instantly. The SEVA platform currently contains nine different replication systems [11]. The first one, the origin of the R6K plasmid, is intended for suicide vectors and requires the π protein for plasmid maintenance usually provided by an appropriate *pir*+ host strain [12]. The remaining eight replication systems are either broad host range systems (2, RK2; 3, pBBR1; 4, pRO1600/ColE1; 5, RSF1010) or specific for enteric bacteria (6, p15A; 7, pSC101; 8, pUC; 9, pBR322). The genetic structure and mode of replication heavily differs between replication systems. For instance, the *oriV*s of pSC101 and RK2 carry typical short direct repeats for binding of a plasmid-encoded single Rep protein, while RSF1010 carries no less than three *rep* genes encoding proteins for priming, unwinding of DNA, and initiation of replication (*repA, repB, repC*. Further mobility related ‘*mob*’ genes have been deleted in the SEVA version). ColE1-like origins including pUC, pBR322 (also called pMB1) and the more distant p15A origin carry no *rep* genes at all and use two sense/antisense RNAs for priming the replication process [2]. The pBR322 origin is the prototype of the class while the pUC origin carries a point mutation in the sense RNA that stabilizes the priming complex, resulting in higher copy number [13]. Stated average copy numbers for these replication systems are often only rough estimates, e.g. RK2 is regarded as a very ‘low-copy’ vector while pBBR1 is ‘medium-copy’ [1]. Such estimates were often obtained by semi-quantitative gel electrophoresis [14–16] or, more accurately, by quantitative real-time PCR (qRT-PCR) [17–19]. However, qRT-PCR strongly depends on the calculation of PCR efficiency to obtain reliable copy numbers.

According to the SEVA nomenclature, replication systems take up the second position in the three digit code (e.g. 2 in ‘pSEVA123′), while the first and the third represent the antibiotic resistance and the cargo genes, respectively. Here, we used the Kanamycin resistance gene and a *styA*-*EGFP styB* (*AEB*) expression cassette as a cargo module that was already described previously in studies using *Pseudomonas* [7, 20]. This cassette consists of a styrene monooxygenase (*styA*) in frame fused to an *EGFP* reporter and an FAD cofactor reductase (*styB*). The original purpose of this construct is the conversion of styrene to styrene oxide and it was used here as a realistic model of a biotechnological process. However, problems related to low PCN and plasmid loss in a part of the population were the starting point for the systematic investigation of plasmid stability in this study. Our aim was to determine the PCN of standard plasmid vectors by highly accurate Droplet Digital PCR (ddPCR), and to reveal relationships between copy number and the degree of heterogeneity as determined by EGFP fluorescence. While fluorescence can be measured on the level of single cells using flow cytometry, determination of PCN by ddPCR requires a larger cell sample. The method applied here employed cell sorting followed by ddPCR and was previously tested with cell numbers ranging from 1 to 10,000 [7]. It appeared that the PCN of a sub-population was constant, regardless how many cells were used, but cell numbers of 1 or 10 showed very high variation and were not suitable. We therefore decided to use 1000 sorted cells of a single, selected sub-population to determine PCN, a number that yielded the best performance in ddPCR (low variation and optimal droplet occupation) [7].

## Results

### Cloning of p2X4-AEB vector series

**Fig. 1:**
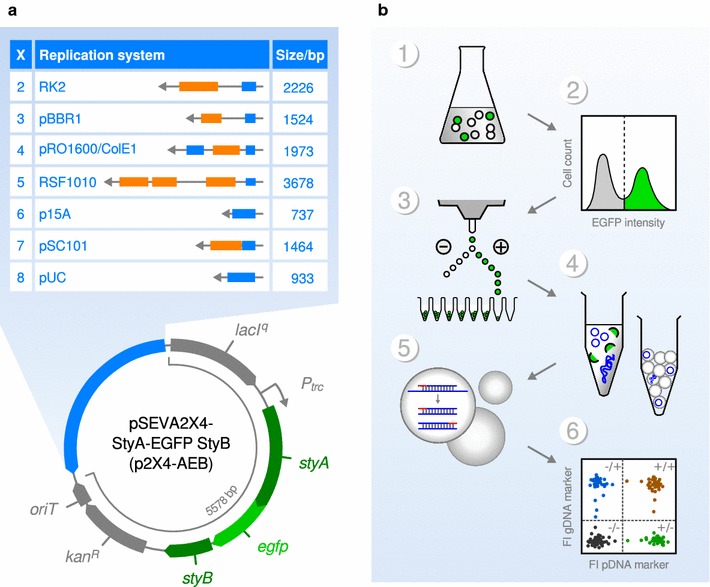
Design of plasmid vectors and experimental strategy. **a** Genetic map of p2X4-AEB plasmid vector series. Seven different replication systems (X) obtained from the SEVA repository were cloned into a typical expression vector, pSEVA2X4-StyA-EGFP StyB (p2X4-AEB). Listed in the table is the official SEVA module number (2–8), name and structure of the replication system (orange—replication initiation proteins, blue—origin of replication), and size. The map illustrates the genetic structure of the plasmid backbone including the *lacI* ^*q*^ repressor, the *tryp*-*lac* hybrid promoter (*P*_*trc*_), a styrene monooxygenase (*styA*) in frame fused to an *EGFP* reporter, an FAD cofactor reductase (*styB*), a kanamycin resistance gene (*kan* ^*R*^) and the origin of transfer (*oriT*). bp—base pairs. **b** Cell sorting and digital PCR for copy number determination. 1—bacterial cultivation, 2—population analysis by flow cytometry, 3—sorting 1000 cells/well, 4—DNA extraction and droplet formation, 5—Droplet Digital PCR reaction, 6—counting positive and negative droplets. Adapted with permission from [7]. Copyright (2014) American Chemical Society

We used the SEVA platform to create a range of new plasmid vectors being completely identical except for the replication system [11]. Of the nine replication systems in the SEVA repository, we omitted the first one, R6K, as it is intended for suicide vectors, and the last one, pBR322. The pBR322 origin is already present in system four (the combined origins of plasmid pRO1600 and ColE1) and very similar to system eight (pUC). The remaining replication systems two to eight were cloned in a plasmid series featuring a Kanamycin resistance cassette and the IPTG inducible *lacI* ^*q*^ repressor/*P*_*trc*_ promoter system that is readily available in the SEVA repository. These seven plasmids were named pSEVA2X4 (abbreviated p2X4), where X represents one of the seven replication systems (2–8). As a readout for gene expression, the *styA*-*EGFP styB* cassette (*AEB*) was cloned under the control of the *P*_*trc*_ promoter allowing measurement of induced fluorescence and these vectors were named p2X4-AEB accordingly (Fig. 1).

### Induction of gene expression and population heterogeneity

**Fig. 2:**
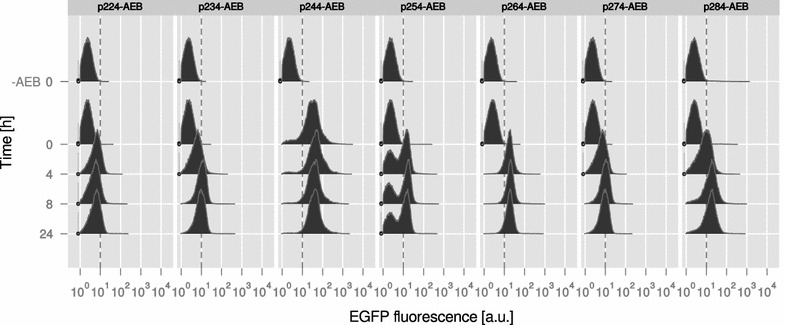
EGFP intensity of seven plasmid-carrying *E. coli* strains. The p2X4-AEB vector series was analyzed via flow cytometry to evaluate productivity and population heterogeneity in *E. coli* DH5α λ*pir*. Representative samples were taken from a batch cultivation for 24 h with induction by IPTG after 2 h. The *first row* (-AEB) shows no-fluorescence control strains carrying the p2X4 plasmid series devoid of *styA*-*EGFP styB*. a.u.—arbitrary units, dashed line—threshold used for discrimination of EGFP negative and positive cells

**Fig. 3:**
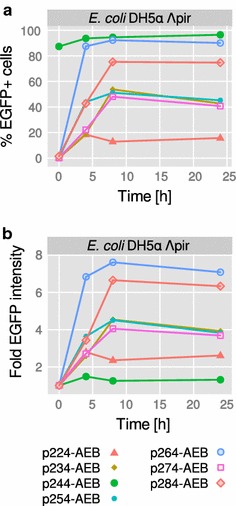
Quantitative analysis of flow cytometry measurements. The p2X4-AEB vector series was analyzed in *E. coli* DH5α λ*pir* as described in Fig. 2. **a** Percent EGFP positive cells when using the threshold shown in Fig. 2 at an EGFP intensity of 10^1^. **b** Fold change in median EGFP intensity of the total populations shown in Fig. 2. Reference is the 0 h time point

The first aim was to compare general characteristics of these plasmids regarding growth, inducibility, and fluorescence yield in a standard bacterial strain, here *E. coli* DH5α carrying a λ*pir* prophage [21]. To this end, *E. coli* was transformed with the seven different expression plasmids (p2X4-AEB) and batch cultivated for 24 h in minimal medium, with and without IPTG induction (1 mM) after 2 h of cultivation (Additional file 3: Figure S1A). The maximum growth rate (µ_max_) ranged from 0.43 to 0.55 h^−1^, and no significant differences were observed between cells carrying the different plasmids or between induced and non-induced conditions. This observation was consistent with results from an independent reproduction (Additional file 3: Figure S1B), and also with the finding that control strains carrying plasmids without the reporter gene cassette (*E. coli* DH5α p2X4) showed a similar growth rate to the strains carrying the full constructs (Additional file 3: Figure S1C). In the next step, flow cytometry was chosen as a sensitive method to analyze EGFP fluorescence on the single cell level. To this end, the seven p2X4-AEB carrying *E. coli* strains were analyzed at four different time points (0, 4, 8, 24 h), in addition to seven *E. coli* p2X4 strains not carrying *styA*-*EGFP* as a negative control (Fig. 2). No fluorescence was detected for all p2X4 strains at 0 h (equal to the 24 h time point of the pre-cultivation), while p2X4-AEB strains showed increasing fluorescence after induction (4–8 h). However, not all strains showed the expected induction pattern. For instance, *E. coli* p244-AEB showed high initial fluorescence with more than 80% of the cells being fluorescent already at 0 h (Fig. 3a). Another strain, *E. coli* p254-AEB, was split in two distinct sub-populations of low and high EGFP fluorescence. In contrast, the *E. coli* strains carrying plasmids p224-, p234-, p264- and p274-AEB showed a normal distribution of EGFP intensity and no fluorescence before induction (Fig. 3a). When comparing EGFP intensity during the course of the cultivation, the mean fluorescence of the population increased between 1.5-fold for p244-AEB and eightfold for p264-AEB (Fig. 3b). The highest absolute EGFP intensity was recorded for p244-AEB after 4 h of induction (median = 44.9) followed by p264 and p284 with 19.7 and 16.9 after 8 h, respectively. The other strains reached maximum intensities between 5.3 and 10.5 (Additional file 1). These observations were verified by independent reproduction of the experiment, with the notable exception of *E. coli* p284-AEB showing a higher initial fluorescence similar to *E. coli* p244-AEB (Additional file 3: Figure S2).

### Average PCN by Droplet digital PCR

**Fig. 4:**
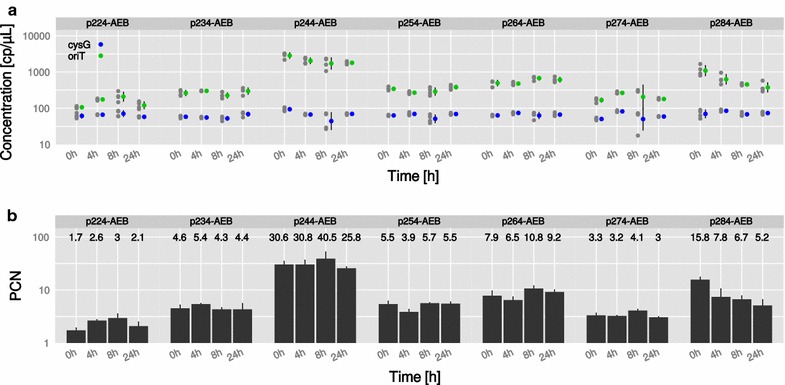
Average plasmid copy number (PCN) of p2X4-AEB vectors in *E. coli*. **a** Absolute concentration of the gDNA and pDNA marker genes *cysG* and *oriT* as determined by Droplet Digital PCR. Representative samples were taken from a batch cultivation for 24 h with induction by IPTG after 2 h. *Grey dots* single measurements, *colored dots* mean and standard deviation of four replicates. **b** PCN calculated as the ratio of pDNA (*oriT*) concentration and gDNA (*cysG*) concentration. *Bars* represent mean and standard deviation

The seven pSEVA plasmids only differing in the replication system demonstrated different degrees of heterogeneity in terms of EGFP fluorescence. One explanation for such differences can be the average copy number of the plasmid, with smaller PCNs being more likely to create plasmid-free sub-populations [4, 5]. Therefore, the average PCN of all seven plasmid-bearing *E. coli* strains was determined by Droplet Digital PCR (ddPCR) according to a recently published workflow (Fig. 1b) [7]. For this purpose, 1000 cells per sample were sorted into microwells by flow cytometry, heat treated to extract DNA, and used as template for a duplex ddPCR reaction. This reaction was targeted at two genetic markers, *oriT*, the *origin of transfer* as a universal marker for all SEVA plasmids, and *cysG*, encoding a siroheme synthase, as a single-copy genomic reference gene previously used in qRT-PCR [22]. The PCN was then calculated as the ratio of plasmid DNA and genomic DNA concentration (*c*_*pDNA*_ */c*_*gDNA*_). The determined PCN was similar for the four time points of each strain (0, 4, 8, 24 h), but showed higher variation between strains (Fig. 4). The lowest and highest PCN found for a single sample were 1.7 and 40.5 for p224-AEB (0 h) and p244-AEB (8 h), respectively. The average PCN across all time points for plasmids p224-AEB to p284-AEB was 2.4 ± 0.6, 4.7 ± 0.7, 31.9 ± 8.8, 5.1 ± 0.9, 8.6 ± 1.9, 3.4 ± 0.5 and 8.9 ± 4.5. An independent reproduction of the experiment mainly confirmed these results with the exception of plasmids p264- and p284-AEB, which had a higher average PCN of 11.6 ± 3.0 and 15.1 ± 4.6, respectively. (Additional file 3: Figure S3).

### PCN of heterogeneous populations

**Fig. 5:**
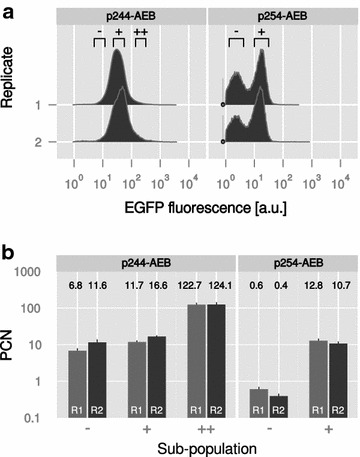
Plasmid copy number (PCN) of selected sub-populations. Two *E. coli* strains with a different degree of heterogeneity regarding EGFP intensity were used for further characterization of PCN. **a** For *E. coli* DH5α λ*pir* p244-AEB, three sub-populations of low (−), intermediate (+) and high EGFP fluorescence (++) were chosen. For *E. coli* DH5α λ*pir* p254-AEB, two sub-populations were chosen according to a low (−) and a high EGFP intensity peak (+). For ddPCR, 1000 cells of each sub-population were sorted. **b** PCN of sorted sub-populations as described before. R1, R2—independent biological replicates. *Bars* represent mean and standard deviation

However, average copy numbers can strongly obscure the real situation, as the characteristics of all cells across the population are summarized. For example, a population can be split into two extreme conditions, plasmid-bearing and plasmid-free, while the average PCN will represent neither condition accurately [7]. Therefore, we chose two strains of *E. coli*, one normally distributed strain (p244-AEB) and one with a bimodal distribution of EGFP fluorescence (p254-AEB), and determined the PCN of selected sub-populations. For the first strain with plasmid p244-AEB, three sub-populations were chosen that represented cells with low (−), intermediate (+) and high EGFP fluorescence (++) as measured by flow cytometry (Fig. 5a; Additional file 1). The first (−) and the last sub-population (++) represented the tails with a proportion of 2.7–3.0 and 0.9–1.8% for two independent replicates, respectively. The intermediate sub-population (+) represented the peak of the distribution (31.5–36.8%). The gating scheme for the second strain carrying p254-AEB comprised two sub-populations, a non-fluorescent (−) and a fluorescent one (+) corresponding to 21.9–24.6 and 38.6–39% of the total population. The PCN of sub-populations was determined via cell sorting and ddPCR in the same manner as for the average populations before and showed remarkable differences (Fig. 5b). For p244-AEB, the intermediate sub-population (+) contained up to 16.6 copies, while the non-fluorescent (−) and highly fluorescent (++) sub-populations had a mean PCN of 9.2 ± 3.4 and 123.4 ± 1.0, respectively. The (−) and (+) sub-populations of p254-AEB were markedly different as well with a mean PCN of 0.5 ± 0.1 and 11.8 ± 1.5, respectively.

## Discussion

### Replication systems associated with population heterogeneity

In this study, we determined the plasmid copy number and marker gene expression for a set of seven standardized SEVA plasmids equipped with different replication systems and a *styA*-*EGFP* reporter. We found that induction of gene expression was possible using a standard concentration of 1 mM IPTG, and that induction posed no additional burden to the cells regarding the growth rate of the strains. The maximum induction in terms of EGFP expression did not exceed an eightfold increase in fluorescence measured for p264-AEB via flow cytometry. This corresponds to publications reporting lower gene expression levels for the *lacI* ^*q*^ */P*_*trc*_ system encoding the ‘quantitative’ LacI repressor compared to the native LacI repressor [23].

However, flow cytometry revealed a variable degree of heterogeneity regarding *styA*-*EGFP* expression for the seven plasmids. There was clearly a group of plasmids giving rise to strains with a very homogeneous population, namely p224-, p234-, p264- and p274-AEB (RK2, pBBR1, p15A, pSC101). But with the exception of p15A, cells carrying these vectors also showed the lowest final EGFP intensity (Fig. 3b; Additional file 1). This is contrasted by the strains carrying p244- and p284-AEB (pRO1600/ColE1, pUC), showing the highest EGFP expression (median fluorescence of 44.9 and 16.9, respectively), together with p264-AEB (19.7). But in terms of heterogeneity, the two strains p244- and p284-AEB also exhibited a more tailed distribution of the population. A similarity of the plasmids p244 and p284 can be expected as they are genetically closely related by sharing a ColE1 type replication origin. In addition, plasmid p244-AEB and, in a reproduction of the experiment, also p284-AEB showed strong initial fluorescence before induction combined with low inducibility. A completely different picture on the single cell level was seen for p254-AEB (RSF1010), which was split into two sub-populations of almost equal proportion (51% EGFP positive at 8 h, Fig. 3a). Such a bimodal distribution can be related to feed-forward regulation of gene expression, where transcription of a gene is activated by its own product [24], but also to a variable gene dosage per cell. The latter would mean that two sub-populations with differential PCN exist or that one sub-population has entirely lost the plasmid, as was already shown for *Bacillus megaterium* [25] and *Pseudomonas putida* [7].

### Population heterogeneity was not correlated to average PCN

A common hypothesis is that a low average PCN leads to higher cell-to-cell variability of PCN and thus to a higher degree of heterogeneity regarding expression of plasmid-encoded genes [4, 5]. Here, we determined the PCN of total populations in a robust and highly accurate manner and compared these to the degree of heterogeneity in EGFP fluorescence. The obtained average copy numbers ranged between 2 and 40, but lower PCNs were clearly dominant. If the seven different replication systems are arranged in groups of low copy (PCN of 1-10), medium copy (PCN of 10-20) or high copy number (PCN of 20-100), plasmids p224-, p234-, p254- and p274-AEB (RK2, pBBR1, RSF1010, pSC101) fall into the first category. Plasmids p264- and p284-AEB (p15A, pUC) showed a higher PCN of up to 15, falling into the second category, while p244-AEB (pRO1600/ColE1) was the only plasmid reaching consistently high copy numbers. If these results are compared with PCN values reported in literature, the copy numbers determined in this study remain at the lower end of the given ranges.

For instance, RK2 is a known low-copy replicon reported to have three to seven copies per chromosome [26], but we found an even lower average PCN of two. The pBBR1 replicon was reported to have five to ten copies per chromosome/cell in *E. coli* [27], while we determined an average PCN of five. The combined replication system pRO1600/ColE1 yielded a PCN of up to 40, which is in accordance with previous reports ranging between 15 and 50 copies per cell for the ColE1 origin [28, 29]. We presume that the pRO1600 origin on its own is not functional in *E. coli* DH5α, as it is dedicated to *Pseudomonas,* where pRO1600/ColE1 shuttle vectors reach average copy numbers between 1 and 7 depending on the host strain [7, 20]. However, the pUC origin, which deviates from ColE1 only by a single base pair, yielded lower copy numbers, although it was reported to reach very high copy numbers in *E. coli* up to several hundred [13, 17]. One reason for the lower copy number of pUC may lie in a higher level of heterogeneity known for such copy-up mutations, as they cause a mis-regulation of the fine-balanced negative feedback loop [30]. More precisely, stronger binding of the primer RNA (RNA II) by copy-up mutations leads to higher frequency of replication initiation while the level of inhibitory antisense RNA (RNA I) remains the same. This promotes a positive feedback loop that can lead to strongly increased PCN for some cells, but to lower PCN for others that are unable to cope with ‘runaway’ plasmid replication [30]. Eventually the average PCN is reduced after prolonged cultivation. Another possibility is the compensation of the copy-up mutation by secondary ‘suppressor’ mutations [31]. The appearance of such mutations during cultivation is evolutionarily favored by reducing the metabolic burden arising from plasmid replication and recombinant gene expression. However, we did not test this hypothesis by sequencing the plasmid during or after cultivation. The copy numbers of plasmids containing the RSF1010 and pSC101 replicons (p254-AEB, p274-AEB) were reported to be around 11.2 and 4.2 per chromosome in *E. coli* [32], respectively. This is higher than the average PCN of 5.2 and 3.4 we determined here, but still in a similar range. This was also true for the best inducible and most homogeneous *E. coli* strain carrying the p15A replicon (p264-AEB) with an average PCN of 8.6 compared to previously reported copy numbers of 14–16 [33].

The question arises, why the PCNs determined here are generally lower than the ones reported in previous studies. As this effect is consistent throughout all tested constructs, it is most likely related to factors of the *E. coli* host strain, the cultivation conditions, method of PCN determination, or elements of the plasmid backbone. It is, for example, a known fact that some strains maintain the same plasmids at higher copy numbers than others. The *E. coli* DH5α derivative used in this study belongs to the K12 family that can deviate in its PCN from other *E. coli* families, such as the B strains, or show variable PCN within strains of the same family [34]. It was also demonstrated that PCN can depend on growth rate [35, 36], but this seemed to have a minor effect here, as only small differences were found across the logarithmic and stationary growth phases. Probably more important was the effect that the average PCN is negatively correlated to the size of the plasmid backbone [30, 37], which here amounted to 5578 bp (without replication system) for the p2X4-AEB vectors and was therefore larger than an EGFP-only reporter construct (~3700 bp). Interestingly, another discussed factor is the metabolic burden from protein production, but no significant effect was observed when comparing PCN before and after induction. Furthermore, the method of PCN determination can yield very different results depending on the reference that is chosen. The PCN in this study was calculated as the ratio of plasmid copies to chromosomal copies, which is a more conservative estimation than using plasmid copies per cell. For example, a cell with ten plasmid copies and two chromosomal copies will have a PCN of five related to a genomic reference gene but a PCN of ten related to the single cell. The copy number of the reference gene *cysG* varied between 50 and 100 cp/µL (Fig. 4a; Additional file 4) corresponding to 1–2 cp/sorted cell (see “Methods” section). For example, a twofold higher PCN can be computed for cells with two chromosomal copies when cell number is used as reference. Cell number is often used as reference when methods other than qPCR are applied [26–29, 33], yielding a higher average PCN simply because there is no standardized definition of PCN.

### Averaged copy numbers do not represent the PCN distribution in situ

The analysis of EGFP fluorescence of the population via flow cytometry revealed a bimodal distribution of cells carrying plasmid p254-AEB (RSF1010). This was compared to a plasmid with a unimodal distribution of the population, p244-AEB (pRO1600/ColE1). Selected sub-populations of *E. coli* carrying either of the two plasmids indeed showed strong differences in PCN corresponding to EGFP intensity. The PCN of the highly fluorescent sub-populations met or exceeded average copy numbers indicated in the literature (11.2 compared to 10–12 for RSF1010 [32], >100 compared to 50 for ColE1 [29]), while the other sub-populations showed a lower PCN. In fact, the almost plasmid-free sub-population (PCN = 0.5) in *E. coli* DH5α p254-AEB constituted at least 22% of the total population and thus strongly reduced the average PCN. Even more remarkable was the wide range of PCN found in the unimodal population of p244-AEB, spanning more than one order of magnitude (9–123). Moreover, the PCN of the central sub-population (‘ + ’) was twofold lower than the average PCN of the same strain (12–17 compared to 32). Apparently, the average PCN is biased by a small fraction of cells, particularly at the tail of the EGFP intensity distribution, that bear an extremely high number of plasmids. This gives rise to the assumption that other strains with unimodal populations have a similar degree of underlying heterogeneity. We therefore argue that the average PCN does not represent the true distribution of PCN in a population.

A known cause for this cell-to-cell variability of PCN is unequal distribution of plasmids caused by imperfect partitioning to daughter cells, a stochastic process especially affecting low-copy-number plasmids [4, 5]. However, the low-copy, broad-host-range replicon RSF1010, originally isolated from *E. coli* [38], was not known to be associated with heterogeneity until now. And replication systems with an even lower average PCN (RK2, pBBR1, pSC101) did not show any bimodality under the same conditions in this study. The instability of the RSF1010-based plasmid is presumably not related to the low average PCN but to interferences between replication system RSF1010 and host factors in *E. coli* DH5α. But even high-copy-number plasmids can be prone to unequal partitioning, for example owing to attachment of plasmid clusters at one cell pole, as demonstrated in *Bacillus megaterium* [25] or clustering of plasmids in foci at different positions in the cell as shown for a pUC19 derivative in *E. coli* DH5α [39]. In the latter study, plasmid localization was determined by single cell fluorescence microscopy and the authors in fact found that the majority of plasmids (average PCN of 70) was clustered in only one or two foci which rapidly moved, associated and dissociated. Plasmid clusters act like one plasmid molecule during cell division and can be expected to increase variability and the rate of segregational plasmid loss. Here, a high degree of heterogeneity was indeed observed for the high-copy ColE1 origin, but no complete plasmid loss, as was the case for RSF1010. Higher cell-to-cell variability for ColE1 type plasmids can also be explained by the fine-balanced feedback regulation via sense and antisense-RNAs that is easily disturbed by stress conditions such as recombinant protein production. The molecular mechanism is the increase of unloaded tRNAs during production that can directly bind RNA I or RNA II secondary structures and thus interfere with negative feedback regulation of PCN [30].

## Conclusion

Considering the wealth of available plasmid vectors and their importance in science and industry, only little is known about the average plasmid copy number of a particular replication system, the copy number variability from cell to cell, and the population heterogeneity arising from it. We addressed this question by constructing seven typical expression plasmids only differing in their replication system. The EGFP fluorescence determined on the single cell level allowed us to assess population heterogeneity, which was low for most of the plasmid vectors. Only one plasmid (carrying the RSF1010 replication system) produced a high amount of heterogeneity in terms of a clear bimodality. The PCN of the constructs ranged from low (2) to high (40), but was generally at the lower end of previously reported PCN ranges. No significant change between different time points of growth was observed. Importantly, there was also no relationship between average PCN and the degree of heterogeneity in terms of EGFP expression. This is in contrast to results obtained by Kittleson and colleagues [8] where a higher average PCN correlated with lower variability. However, we further characterized the variability of PCN on the sub-population level for two *E. coli* strains, one carrying a plasmid with the low-copy RSF1010 origin showing a bimodal distribution of EGFP fluorescence, and one with the high-copy ColE1 replication system having a unimodal distribution (mean PCN of 5.2 and 32, respectively). For the unimodal population (ColE1), extreme copy numbers were found in sub-populations representing the tails of the distribution. For the bimodal population (RSF1010), the non-fluorescent sub-population was almost plasmid-free. This allows the conclusion that the average PCN can only be used as a rough approximation. It is not meaningful for heterogeneous (multimodal) populations, and even a unimodal population with normally distributed EGFP intensity may cover a huge spectrum of PCNs as was demonstrated here for the ColE1-based plasmid (9 < PCN < 123).

## Methods

### Bacterial strains and cultivation

All experiments were performed using an *E. coli* DH5α strain carrying a λ prophage with the *pir* gene (λ*pir*) for maintenance of R6K replicons [21], kindly provided by Victor de Lorenzo, CNB-CSIC, Madrid, Spain. Cloning of vectors was performed using *E. coli* DH5α λ*pir* or *E. coli* BL21 (DE3) obtained from the German Collection of Microorganisms and Cell Cultures (DMSZ). For cloning, bacteria were grown in liquid LB medium (5 g/L yeast extract, 5 g/L NaCl, 10 g/L tryptone) or plated on solid LB medium containing 2% (w/v) agarose. Induction experiments were carried out in minimal medium composed of 0.5 g/L MgSO_4_ × 7H_2_O, 0.015 g/L CaCl_2_ × 2H_2_O, 0.5 g/L NaCl, 6 g/L Na_2_HPO_4_ × 2H_2_O, 1 g/L NH_4_Cl, 3 g/L KH_2_PO_4_, 12.5 µM ZnSO_4_ × 7H_2_O, 2.5 µM CuSO_4_ × 5H_2_O, 2.5 µM H_3_BO_3_, 10 µM FeSO_4_ × 7H_2_O, 50 µM CaCO_3_, 12 µM MnSO_4_ × 7H_2_O, 2.5 CoSO_4_ × 7H_2_O, 1 mM Thiamin, and 0.5% w/v glucose as carbon and energy source. Kanamycin was added for plasmid selection at 50 mg/L final concentration. A 5 mL volume of minimal medium in a 50 mL shake flask was inoculated from plate, cultivated overnight at 37 °C with 200 rpm, and used to inoculate a 10 mL volume of minimal medium at an optical density of 0.1 (OD_600 nm_, Ø = 0.5 mm) for cultivation at the same conditions. If required, isopropyl-β-d-thiogalactopyranoside (IPTG) was added for induction at a final concentration of 1 mM. Growth was measured as OD_600 nm_ in transparent 96-well plates filled with 200 µL cell suspension using a Tecan GENios Plus spectrophotometer. For further analysis 500 µL cell suspension were centrifuged for 2 min at 8000×*g* and 4 °C. The supernatant was discarded and the cells re-suspended in 500 µL ice cold cryopreservation buffer as described in [40]. Cell samples were stored at −20 °C until analysis.

### Construction of pSEVA vectors

The construction of vectors was carried out according to standard protocols. The original pSEVA (abbreviated ‘p’) vectors p214, p224, p234, p241, p251, p261, p471 and p281 were obtained from the SEVA repository (http://seva.cnb.csic.es), Madrid, Spain. Cargo module 4 contains the IPTG-inducible repressor/promoter *lacI* ^*q*^ */P*_*trc*_ and was extracted from p214 by *Pac*I/*Avr*II digestion. This 1465 bp fragment was ligated with the *Pac*I/*Avr*II digested vectors p241, p251, p261 and p281 to yield the new vectors p244, p254, p264 and p284. The vector p274 was obtained by extracting the SC101 replication system from p471 using *Fse*I/*Asc*I digestion (1468 bp) and ligating it with the 3020 bp *Fse*I/*Asc*I digested backbone of p244. The *styA*-*EGFP styB* (AEB) insert was obtained by *Eco*RI/*Xma*I digestion of plasmid pA-EGFP_B [7], and inserted in the *Eco*RI/*Xma*I digested pSEVA vectors p224, p234, p244, p254, p264, p274 and p284 to yield the final constructs p224-AEB, p234-AEB, p244-AEB, p254-AEB, p264-AEB, p274-AEB and p284-AEB. For all constructs, positive clones were identified by colony PCR and verified by plasmid isolation, restriction digestion and sequencing of the insert using the proposed SEVA standard primers for T0 and T1 terminators [1]. Plasmids were transferred into *E. coli* strains by electroporation as described [7].

### Flow cytometry and cell sorting

Frozen cell samples were thawed on ice, washed with phosphate buffer (145 mM NaCl, 6 mM Na_2_HPO_4_, 1.8 mM NaH_2_PO_4_, pH 7.2), adjusted to an OD_600 nm_ of 0.05, and filtered by a CellTrics mesh (Partec) with 30 µm pore size. A MoFlo Legacy cell sorter (Beckman-Coulter) equipped with a blue Argon ion laser (Coherent Innova 90C, 400 mW) was used for analysis. Forward scatter (FSC) and side scatter signals (SSC) were acquired using excitation at 488 nm, together with a bandpass filter of 488/10 nm and a neutral density filter of 2.0. EGFP fluorescence was detected in channel FL1 with a bandpass filter of 530/40 nm for emission together with a neutral density filter of 0.3. The alignment of the instrument with fluorescent beads and the sheath buffer composition (here using a twofold dilution) are given in [41]. For acquisition of EGFP intensity, around 50,000 cells of a population were analyzed and a gate at an intensity of FL1 = 10^1^ was used to discriminate between EGFP positive and negative cells. Detailed statistics on flow cytometry samples can be found in Additional file 1. Data acquisition and cell sorting was performed as described in [7]. Briefly, cells and beads (Fluoresbrite^®^ Bright Blue Microspheres, Ø = 0.5 µm, Polysciences) were sorted and deposited in 8-well PCR strips (G003-SF, Kisker Biotech) using the MoFlo’s CyCLONE robotic tray. For each sample, four replicates with 1000 cells or beads per well (equalling 1 µL volume) were sorted at a speed of 100–200 particles/s into 8-well strips pre-filled with 7 µL dH_2_O. The most accurate sorting mode (single cell and one drop mode) was used for highest purity. Cell and bead populations were separated from electronic noise according to the FSC, SSC and FL1 (EGFP) signal intensity. As a control for accurate sorting, a cell sample spiked with beads was used to sort 1000 beads per well as the no-template-control. Sorted samples were immediately stored at −20 °C.

### Sample preparation for ddPCR

DNA was extracted from whole sorted cells by the heat treatment method described in [7]. Sorted cell samples were thawed on ice, heated at 95 °C for 10 min in a Tetrad 2 thermo-cycler (Bio-Rad) and immediately cooled on ice again. The samples were briefly centrifuged at 500×*g* for 3 s to remove residual liquid from tube walls. Different incubation times for heat treatment ranging from 0 to 60 min were tested with ddPCR and the condition with the highest obtained concentration for genomic DNA (10 min) was chosen for further experiments (Additional file 3: Figure S4). For controls, 2 µL of a serially diluted plasmid stock (pA-EGFP_B, 10^9^ copies/µL) or 2 µL of isolated genomic DNA (*E. coli* BL21 (DE3)) was added to the ddPCR mastermix. DNA concentration of stock solutions was determined using a NanoDrop spectrophotometer (Thermo Scientific).

### Primer and probe design

Primers and probes were designed with Primer3 [42] and optimized with PerlPrimer [43] regarding primer dimers, self-priming, melting temperature, aspired G/C content of 30–80% and the presence of GC clamps. Gene sequences were retrieved from http://www.ncbi.nlm.nih.gov and all oligonucleotides were tested for specificity using Primer-BLAST [44]. Designed probes were modified at the 5′ end with the fluorophore FAM for the reference gene *cysG* and HEX for the plasmid marker *oriT,* and furthermore modified at the 3′ end with the quencher BHQ-1. All oligonucleotides were obtained from Eurofins MWG Operon. Probes and primers were tested according to the dMIQE guidelines [45], including optimal concentration, annealing temperature, formation of a single product (using qRT-PCR and gel electrophoresis), and discrimination of negative and positive droplets in ddPCR. A summary of used oligonucleotides is listed in Table 1, the dMIQE checklist is given in Additional file 2.

**Table 1:** Oligonucleotide primers and probes used for ddPCR

Target	Genbank ID	Amplicon length	Primer/probe sequence	T_m_/ °C	Length/bp
*cysG* (*E. coli genome*)	NJ74_RS16775	127	5′-AGCCATTACTGAAACGACC-3′	60.16	19
	[46]		5′-GCTGAATTTGTTGCAGTCC-3′	60.16	19
			5′-FAM-ACCAACCAGCACCACTTCACCG-BHQ-1-3′	69.99	22
*oriT* (*SEVA plasmids*)	JX560321.2	104	5′-CAGGTGCGAATAAGGGAC-3′	60.25	18
	[1]		5′-GTAGACTTTCCTTGGTGTATCC-3′	60.33	22
			5′-HEX-CCTATCCTGCCCGGCTGACG-BHQ-1-3′	69.64	20

### Droplet digital PCR

Droplet digital PCR was performed as described in [7]. Briefly, a duplex reaction set-up was used with simultaneous detection of a reference gene and a target gene. A single reaction volume of 20 µL contained 10 µL 2× ddPCR Supermix (Bio-Rad), 2 µL of primers (final concentration 900 nM) and probes (final concentration 125 nM), and 8 µL template solution. A master mix containing all ingredients except the template was prepared and added to the heat-treated samples in 8-well strips. The samples were thoroughly mixed, briefly centrifuged at 500×*g* for 3 s and transferred to DG8 cartridges (Bio-Rad) for droplet generation with the QX100 system (Bio-Rad) according to the manufacturer. Generated droplets were transferred to a twin.tec 96-well PCR plate (Eppendorf) and sealed for 5 s with a heat sealer (Eppendorf). The PCR reaction was performed in a Tetrad 2 thermo-cycler with the following program: 95 °C for 10 min, 40 cycles of 94 °C for 30 s and 58 °C for 60 s, 98 °C for 10 min, with a ramp rate of 2.5 °C/s. Droplets were analyzed with the QX100 droplet reader with simultaneous detection of FAM and HEX.

### Statistics

Induction experiments were performed with two independent biological replicates, and all PCR experiments were performed with four technical replicates per condition at the stage of cell sorting. Repeatability and inter-assay variation were assessed using the following controls; sorted beads (no-template-control), sorted beads spiked with plasmid DNA (no-gDNA-control), isolated gDNA (no-plasmid-control), and gDNA spiked with plasmid DNA (Additional file 3: Figure S5). Data acquisition for ddPCR was performed with QuantaSoft v1.7 software (calibrated for FAM/HEX) and the four droplet species were manually gated as depicted for the control samples (Additional file 3: Figure S6). Data were exported as text file and further analyzed using R v3.0.2. For each reaction volume of 20 µL up to 15,000 droplets were analyzed with an average droplet volume of 0.85 nL [47]. The PCN was calculated as the ratio of plasmid marker concentration to genomic marker concentration per replicate (*c*_*pDNA*_ */c*_*gDNA*_), indicated is mean and standard deviation of all replicates per condition. For comparison, absolute plasmid copy numbers per cell can be calculated by multiplication of plasmid concentration with the total volume of PCR reaction containing 1000 sorted cells (*PCN*_*per_cell*_ = *c*_*pDNA*_ *·V*_*PCR*_ */*1000). Outliers were not removed except for known pipetting errors. The Droplet Digital PCR dataset is given in Additional file 4.

## Additional files

**Additional file 1.** This spreadsheet file contains statistical information on the flow cytometry data presented in this study.

**Additional file 2.** This spreadsheet file contains the recommended minimal information for digital PCR experiments.

**Additional file 3: Figure S1.** Growth of *E. coli* carrying different plasmids in minimal medium. **A** Comparison of non-induced (0 mM IPTG) and induced (1 mM IPTG) strains of *E. coli* DH5α λ*pir* carrying the seven p2X4-AEB plasmids. Gene expression was induced at 2 h cultivation (dashed line). **B** Independent reproduction of the growth experiment as described in A. **C** Non fluorescent control strains carrying seven p2X4 plasmids without *styA*-*EGFP styB*. Depicted are data from two replicates. OD_600_—optical density at 600 nm, inset numbers, maximum specific growth rate µ_max_. **Figure S2.** Independent reproduction of the experiment shown in Fig. 2. *E. coli* DH5α λ*pir* carrying the p2X4-AEB vector series was analyzed via flow cytometry. Representative samples were taken from a batch cultivation for 24 h with induction by IPTG after 2 h. a.u.—arbitrary units, dashed line—threshold used for discrimination of EGFP negative and positive cells. **Figure S3.** Average plasmid copy number (PCN) of p2X4-AEB vectors in *E. coli*. **A** Absolute concentration of the gDNA and pDNA marker genes *cysG* and *oriT* as determined by Droplet Digital PCR. Representative samples were taken from a batch cultivation for 24 h with induction by IPTG after 2 h. *Grey dots*—single measurements, *colored dots*—mean and standard deviation of four replicates. **B** PCN calculated as the ratio of pDNA (*oriT*) concentration and gDNA (*cysG*) concentration. Bars represent mean and standard deviation. **Figure S4.** Different incubation times for heat treatment ranging from 0 to 60 min were tested with ddPCR. 1000 cells of *E. coli* BL21 (DE3) carrying pA-EGFP_B from a batch cultivation for 2 or 24 h were used as template. **A** Absolute concentration of the gDNA and pDNA marker genes *cysG* and *oriT* as determined by Droplet Digital PCR. The gDNA concentration was highest after 10 min treatment. *Grey dots*—single measurements, *colored dots*—mean and standard deviation of four replicates. **B** PCN calculated as the ratio of pDNA (*oriT*) concentration and gDNA (*cysG*) concentration. Bars represent mean and standard deviation. **Figure S5.** Template controls for ddPCR experiments. Sorted beads (no-template-control), sorted beads spiked with plasmid DNA (no-gDNA-control), isolated gDNA (no-plasmid-control), and gDNA spiked with plasmid DNA were used for simultaneous pDNA (*oriT*) and gDNA (*cysG*) determination. **Figure S6.** Exemplary droplet gating for the four control samples, sorted beads (no-template-control), sorted beads spiked with plasmid DNA (no-gDNA-control), isolated gDNA (no-plasmid-control), and gDNA spiked with plasmid DNA. Black—negative droplets, blue—FAM positive droplets, green—HEX positive droplets, brown—double positive droplets.

**Additional file 4.** This spreadsheet file contains the Droplet Digital PCR dataset presented in this study.

## Abbreviations

CV: coefficient of variation
ddPCR: Droplet Digital PCR
EGFP: enhanced green fluorescent protein
gDNA: genomic DNA
IPTG: isopropyl-β-d-thiogalactopyranosid
oriT: origin of transfer
oriV: (vegetative) origin of replication
PCN: plasmid copy number
pDNA: plasmid DNA
SEVA: Standard European Vector Architecture

## References

[1] Silva-Rocha R, Martínez-García E, Calles B, Chavarría M, Arce-Rodríguez A, de Las Heras A (2013). The Standard European Vector Architecture (SEVA): a coherent platform for the analysis and deployment of complex prokaryotic phenotypes. Nucleic Acids Res.

[2] del Solar G, Giraldo R, Ruiz-Echevarría MJ, Espinosa M, Díaz-Orejas R (1998). Replication and control of circular bacterial plasmids. Microbiol Mol Biol Rev.

[3] Jones KL, Kim SW, Keasling JD (2000). Low-copy plasmids can perform as well as or better than high-copy plasmids for metabolic engineering of bacteria. Metab Eng.

[4] Summers DK (1991). The kinetics of plasmid loss. Trends Biotechnol.

[5] Jahn M, Günther S, Müller S (2015). Non-random distribution of macromolecules as driving forces for phenotypic variation. Curr Opin Microbiol.

[6] Jahn M, Seifert J, von Bergen M, Schmid A, Bühler B, Müller S (2013). Subpopulation-proteomics in prokaryotic populations. Curr Opin Biotechnol.

[7] Jahn M, Vorpahl C, Türkowsky D, Lindmeyer M, Bühler B, Harms H (2014). Accurate determination of plasmid copy number of flow-sorted cells using Droplet Digital PCR. Anal Chem.

[8] Kittleson JT, Cheung S, Anderson JC (2011). Rapid optimization of gene dosage in *E. coli* using DIAL strains. J Biol Eng.

[9] Vilanova C, Tanner K, Dorado-Morales P, Villaescusa P, Chugani D, Frías A (2015). Standards not that standard. J Biol Eng.

[10] Shetty RP, Endy D, Knight TF (2008). Engineering BioBrick vectors from BioBrick parts. J Biol Eng.

[11] Martínez-García E, Aparicio T, Goñi-Moreno A, Fraile S, de Lorenzo V (2015). SEVA, 2.0: an update of the Standard European Vector Architecture for de-/re-construction of bacterial functionalities. Nucleic Acids Res.

[12] Kolter R, Inuzuka M, Helinski DR (1978). Trans-complementation-dependent replication of a low molecular weight origin fragment from plasmid R6K. Cell.

[13] Lin-Chao S, Chen WT, Wong TT (1992). High copy number of the pUC plasmid results from a Rom/Rop-suppressible point mutation in RNA II. Mol Microbiol.

[14] Projan SJ, Carleton S, Novick RP (1983). Determination of plasmid copy number by fluorescence densitometry. Plasmid.

[15] Ryan W, Parulekar SJ (1991). Recombinant protein synthesis and plasmid instability in continuous cultures of *Escherichia coli* JM103 harboring a high copy number plasmid. Biotechnol Bioeng.

[16] Haugan K, Karunakaran P, Tøndervik A, Valla S (1995). The host range of RK2 minimal replicon copy-up mutants is limited by species-specific differences in the maximum tolerable copy number. Plasmid.

[17] Lee CL, Ow DSW, Oh SKW (2006). Quantitative real-time polymerase chain reaction for determination of plasmid copy number in bacteria. J Microbiol Methods.

[18] Carapuça E, Azzoni AR, Prazeres DMF, Monteiro GA, Mergulhão FJM (2007). Time-course determination of plasmid content in eukaryotic and prokaryotic cells using real-time PCR. Mol Biotechnol.

[19] Skulj M, Okrslar V, Jalen S, Jevsevar S, Slanc P, Strukelj B (2008). Improved determination of plasmid copy number using quantitative real-time PCR for monitoring fermentation processes. Microb Cell Fact.

[20] Lindmeyer M, Jahn M, Vorpahl C, Müller S, Schmid A, Bühler B (2015). Variability in subpopulation formation propagates into biocatalytic variability of engineered *Pseudomonas putida* strains. Front Microbiol.

[21] Herrero M, de Lorenzo V, Timmis KN (1990). Transposon vectors containing non-antibiotic resistance selection markers for cloning and stable chromosomal insertion of foreign genes in gram-negative bacteria. J Bacteriol.

[22] Zhou K, Zhou L, Lim QE, Zou R, Stephanopoulos G, Too H-P (2011). Novel reference genes for quantifying transcriptional responses of *Escherichia coli* to protein overexpression by quantitative PCR. BMC Mol Biol.

[23] Balzer S, Kucharova V, Megerle J, Lale R, Brautaset T, Valla S (2013). A comparative analysis of the properties of regulated promoter systems commonly used for recombinant gene expression in *Escherichia coli*. Microb Cell Fact.

[24] Veening J-W, Smits WK, Kuipers OP (2008). Bistability, epigenetics, and bet-hedging in bacteria. Annu Rev Microbiol.

[25] Münch KM, Müller J, Wienecke S, Bergmann S, Heyber S, Biedendieck R (2015). Polar fixation of plasmids during recombinant protein production in *Bacillus megaterium* results in population heterogeneity. Appl Environ Microbiol.

[26] Figurski DH, Helinski DR (1979). Replication of an origin-containing derivative of plasmid RK2 dependent on a plasmid function provided in trans. Proc Natl Acad Sci USA.

[27] Kovach ME, Elzer PH, Hill DS, Robertson GT, Farris MA, Roop RM (1995). Four new derivatives of the broad-host-range cloning vector pBBR1MCS, carrying different antibiotic-resistance cassettes. Gene.

[28] Schmidt L, Inselburg J (1982). ColE1 copy number mutants. J Bacteriol.

[29] Freudenau I, Lutter P, Baier R, Schleef M, Bednarz H, Lara AR (2015). ColE1-plasmid production in *Escherichia coli*: mathematical simulation and experimental validation. Front Bioeng Biotechnol.

[30] Camps M (2010). Modulation of ColE1-like plasmid replication for recombinant gene expression. Recent Pat DNA Gene Seq.

[31] Fitzwater T, Yang YL, Zhang XY, Polisky B (1992). Mutations affecting RNA-DNA hybrid formation of the ColE1 replication primer RNA. Restoration of RNA I sensitivity to a copy-number mutant by second-site mutations. J Mol Biol.

[32] Nagahari K, Tanaka T, Hishinuma F, Kuroda M, Sakaguchi K (1977). Control of tryptophan synthetase amplified by varying the numbers of composite plasmids in *Escherichia coli* cells. Gene.

[33] Hiszczyńska-Sawicka E, Kur J (1997). Effect of *Escherichia coli* IHF mutations on plasmid p15A copy number. Plasmid.

[34] Marisch K, Bayer K, Cserjan-Puschmann M, Luchner M, Striedner G (2013). Evaluation of three industrial *Escherichia coli* strains in fed-batch cultivations during high-level SOD protein production. Microb Cell Fact.

[35] Klumpp S (2011). Growth-rate dependence reveals design principles of plasmid copy number control. PLoS ONE.

[36] Akeno Y, Ying B-W, Tsuru S, Yomo T (2014). A reduced genome decreases the host carrying capacity for foreign DNA. Microb Cell Fact.

[37] Smith MA, Bidochka MJ (1998). Bacterial fitness and plasmid loss: the importance of culture conditions and plasmid size. Can J Microbiol.

[38] Frĕdĕricq P, Krŏmĕry V, Kettner M (1971). Transferable colicinogenic factors as mobilizing agents for extrachromosomal streptomycin resistance. Z Für Allg Mikrobiol.

[39] Pogliano J, Ho TQ, Zhong Z, Helinski DR (2001). Multicopy plasmids are clustered and localized in *Escherichia coli*. Proc Natl Acad Sci USA.

[40] Jahn M, Seifert J, Hübschmann T, von Bergen M, Harms H, Müller S (2013). Comparison of preservation methods for bacterial cells in cytomics and proteomics. J Integr Omics.

[41] Koch C, Günther S, Desta AF, Hübschmann T, Müller S (2013). Cytometric fingerprinting for analyzing microbial intracommunity structure variation and identifying subcommunity function. Nat Protoc.

[42] Rozen S, Skaletsky H (2000). Primer3 on the WWW for general users and for biologist programmers. Methods Mol Biol.

[43] Marshall OJ (2004). PerlPrimer: cross-platform, graphical primer design for standard, bisulphite and real-time PCR. Bioinformatics.

[44] Ye J, Coulouris G, Zaretskaya I, Cutcutache I, Rozen S, Madden TL (2012). Primer-BLAST: a tool to design target-specific primers for polymerase chain reaction. BMC Bioinform.

[45] Huggett JF, Foy CA, Benes V, Emslie K, Garson JA, Haynes R (2013). The digital MIQE guidelines: minimum information for publication of quantitative digital PCR experiments. Clin Chem.

[46] Song Y, Lee B-R, Cho S, Cho Y-B, Kim S-W, Kang TJ (2015). Determination of single nucleotide variants in *Escherichia coli* DH5α by using short-read sequencing. FEMS Microbiol Lett..

[47] Corbisier P, Pinheiro L, Mazoua S, Kortekaas A-M, Chung PYJ, Gerganova T (2015). DNA copy number concentration measured by digital and droplet digital quantitative PCR using certified reference materials. Anal Bioanal Chem.

