# Supplementary figures and images for: Copy number variability of expression plasmids determined by cell sorting and Droplet Digital PCR
Source: Microb Cell Fact. 2016 Dec 19;15:211. doi: 10.1186/s12934-016-0610-8 (PMC5168713; doi:10.1186/s12934-016-0610-8)

Figure S1

A

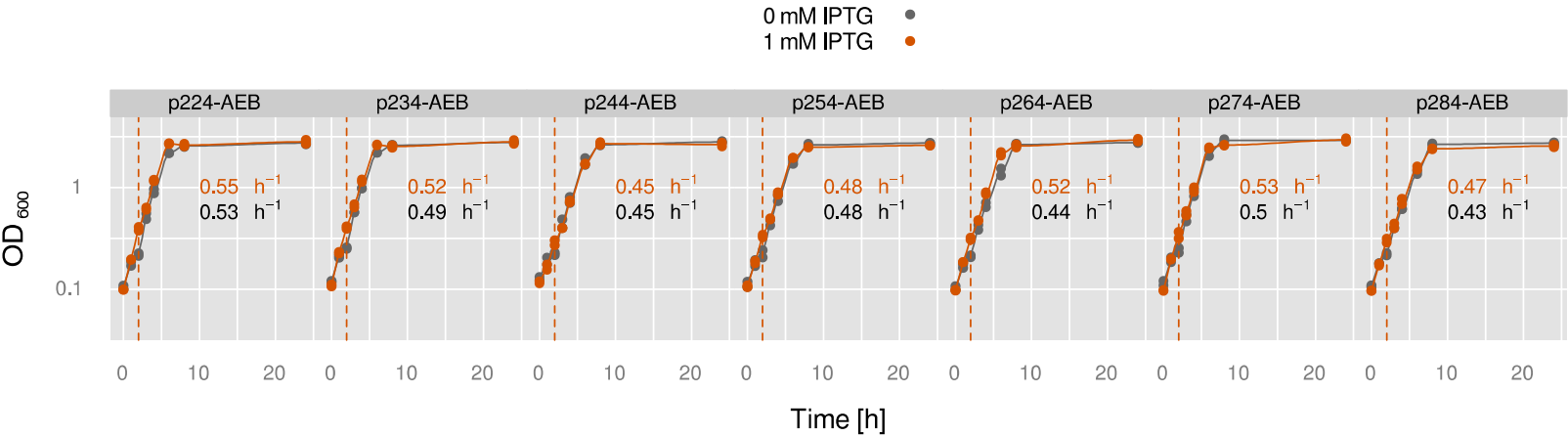

B

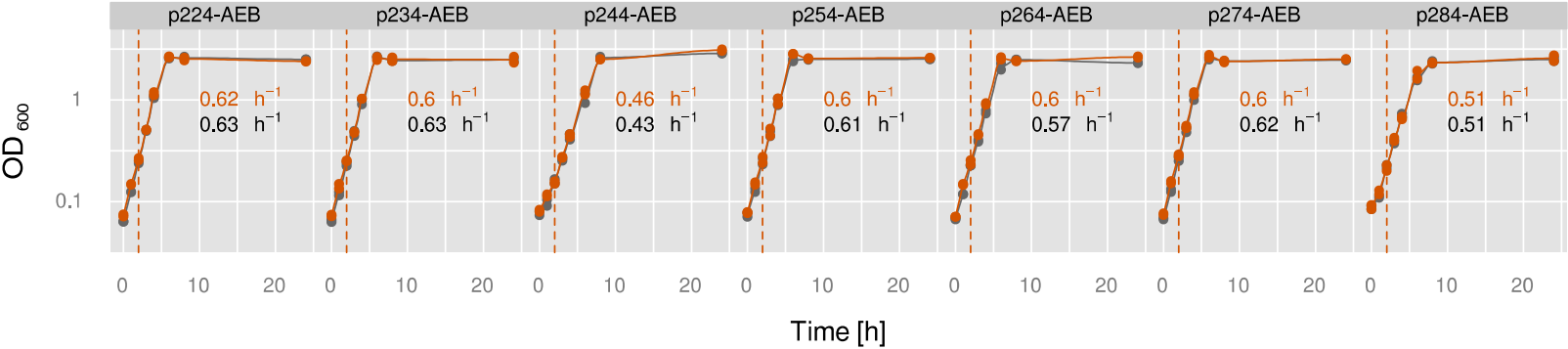

C

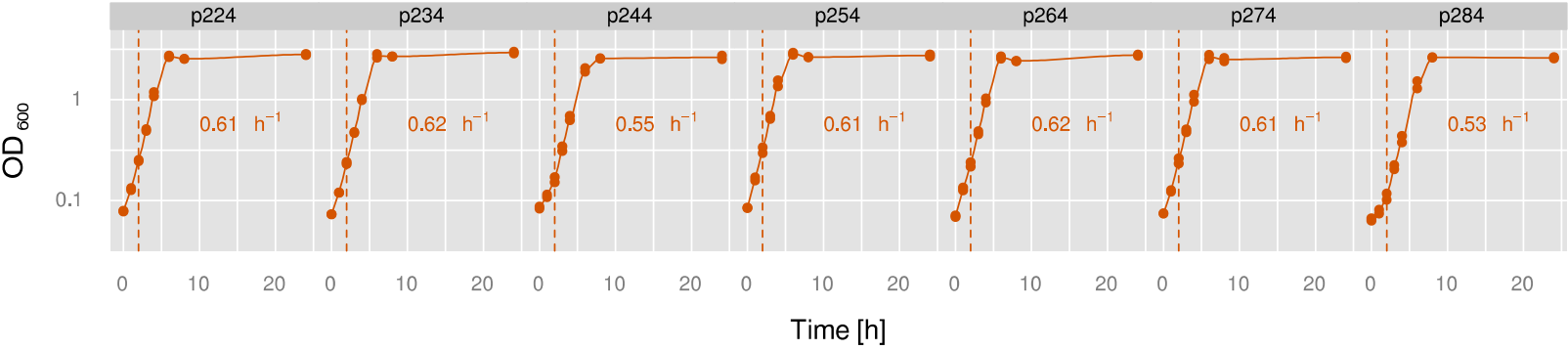

Figure S2

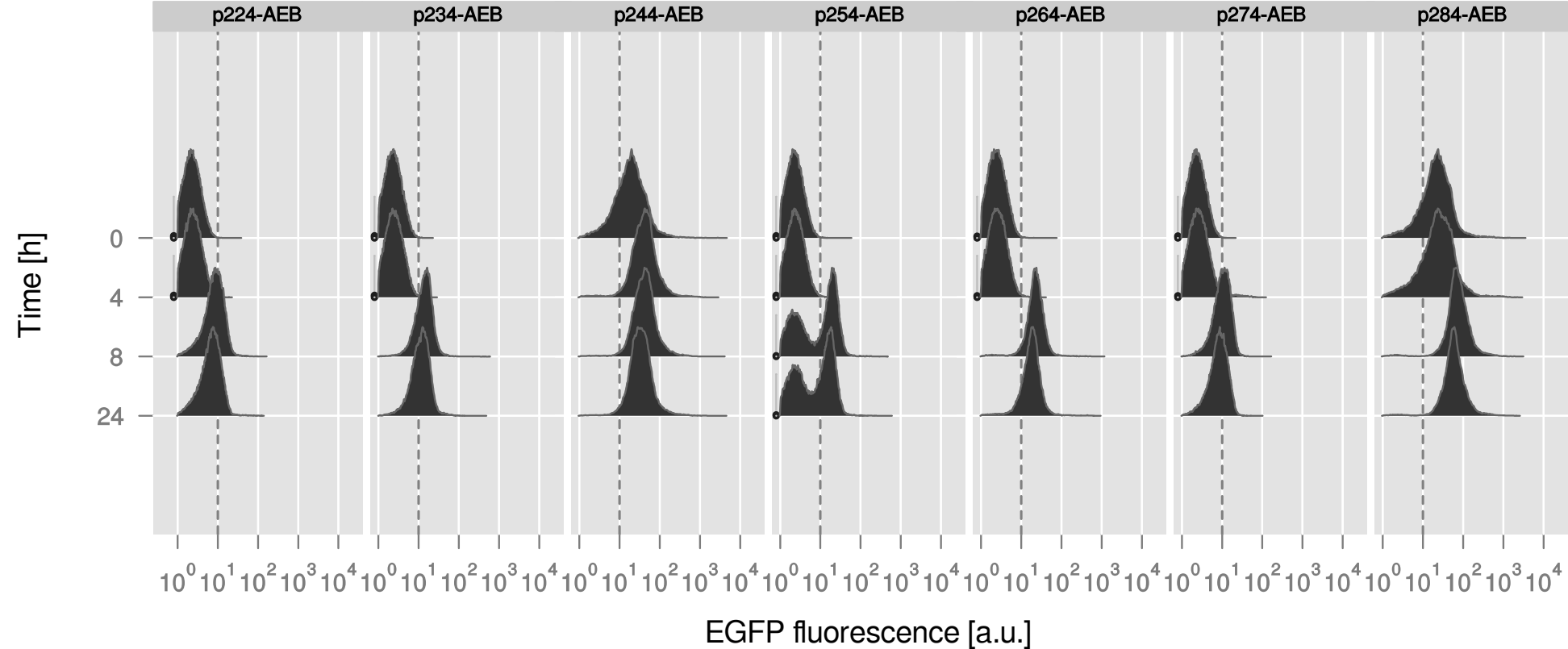

Figure S3

A

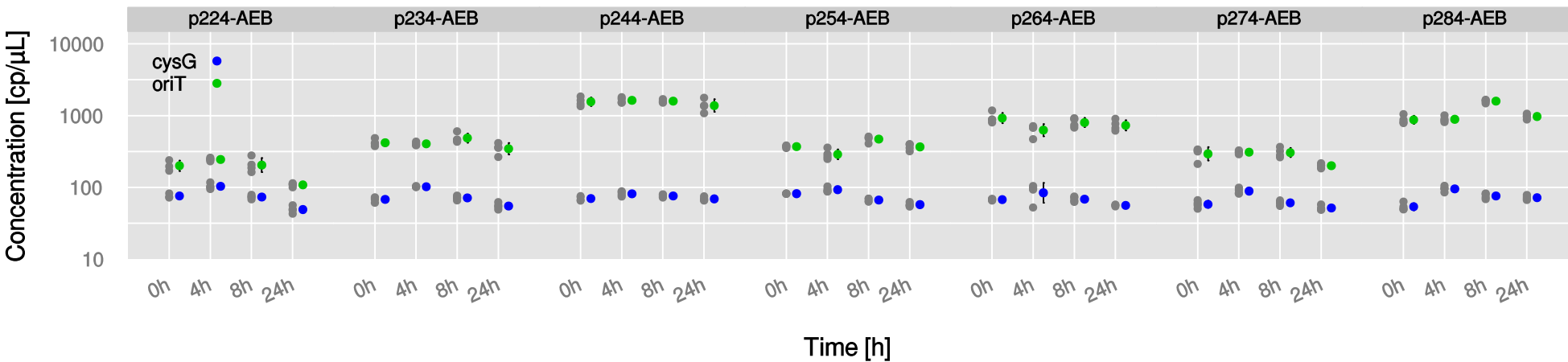

B

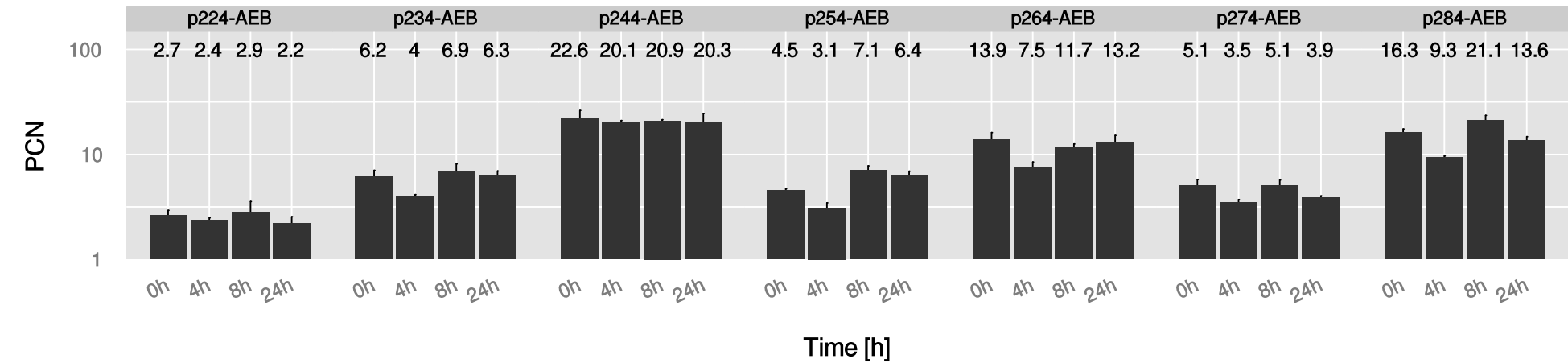

Figure S4

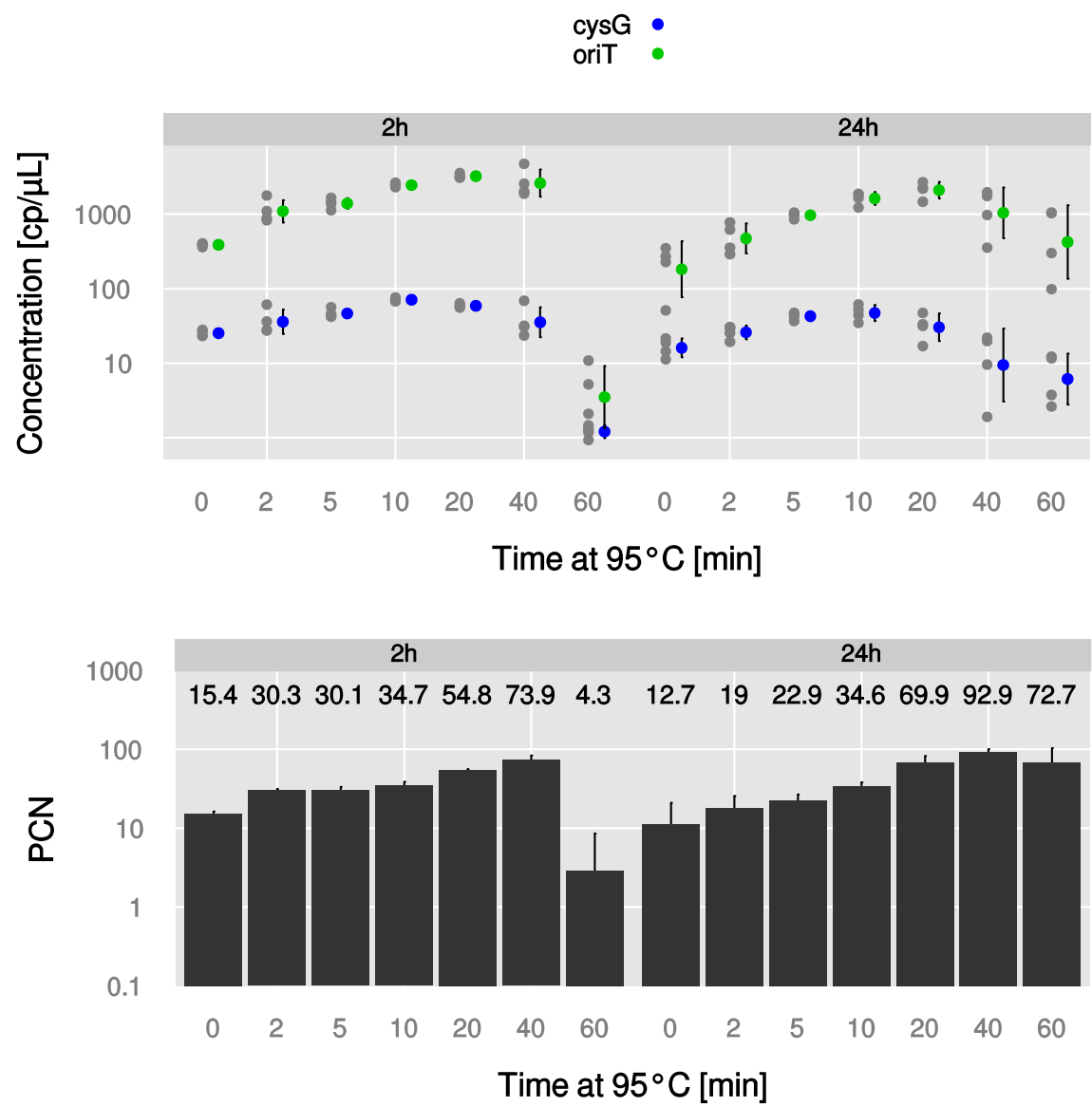

Figure S5

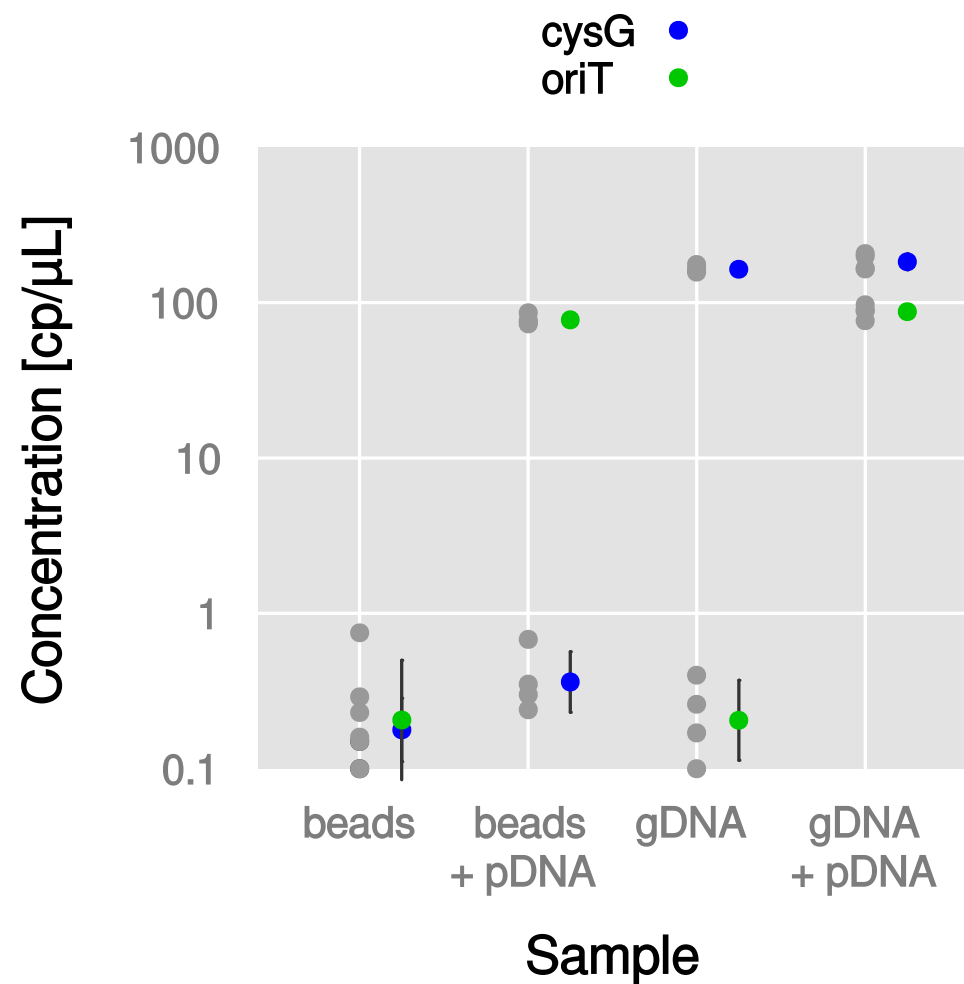

Figure S6

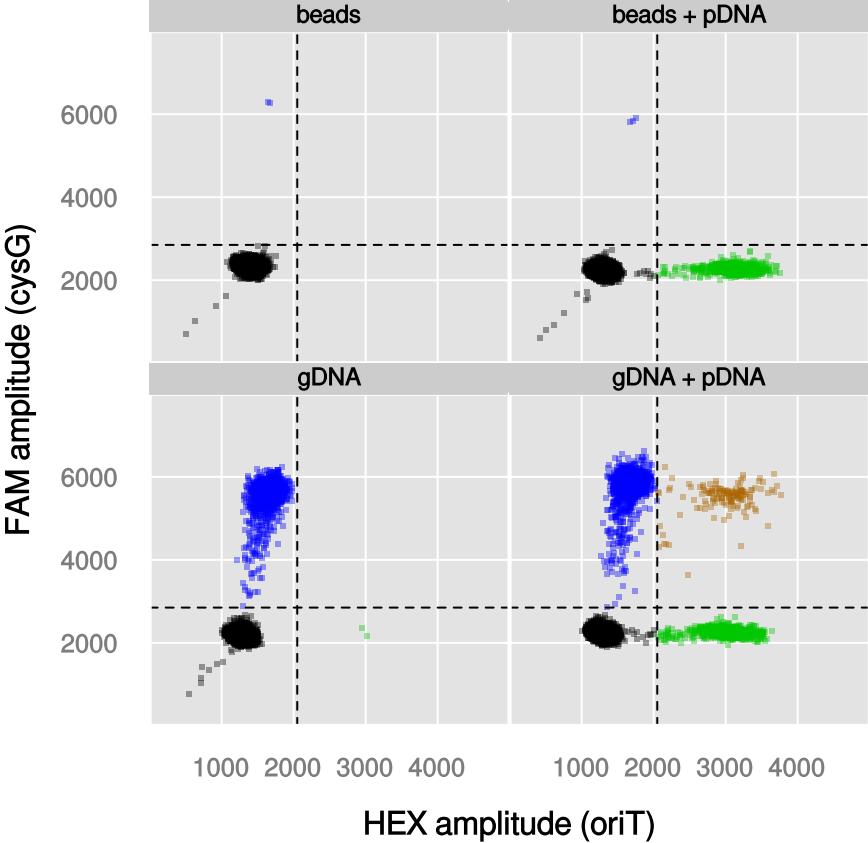

Supplement: Supplementary file 3 — Additional file 3: Figure S1. Growth of E. coli carrying different plasmids in minimal medium. A Comparison of non-induced (0 mM IPTG) and induced (1 mM IPTG) strains of E. coli DH5α λpir carrying the seven p2X4-AEB plasmids. Gene expression was induced at 2 h cultivation (dashed line). B Independent reproduction of the growth experiment as described in A. C Non fluorescent control strains carrying seven p2X4 plasmids without styA-EGFP styB. Depicted are data from two replicates. OD600—optical density at 600 nm, inset numbers, maximum specific growth rate µmax. Figure S2. Independent reproduction of the experiment shown in Fig. 2. E. coli DH5α λpir carrying the p2X4-AEB vector series was analyzed via flow cytometry. Representative samples were taken from a batch cultivation for 24 h with induction by IPTG after 2 h. a.u.—arbitrary units, dashed line—threshold used for discrimination of EGFP negative and positive cells. Figure S3. Average plasmid copy number (PCN) of p2X4-AEB vectors in E. coli. A Absolute concentration of the gDNA and pDNA marker genes cysG and oriT as determined by Droplet Digital PCR. Representative samples were taken from a batch cultivation for 24 h with induction by IPTG after 2 h. Grey dots—single measurements, colored dots—mean and standard deviation of four replicates. B PCN calculated as the ratio of pDNA (oriT) concentration and gDNA (cysG) concentration. Bars represent mean and standard deviation. Figure S4. Different incubation times for heat treatment ranging from 0 to 60 min were tested with ddPCR. 1000 cells of E. coli BL21 (DE3) carrying pA-EGFP_B from a batch cultivation for 2 or 24 h were used as template. A Absolute concentration of the gDNA and pDNA marker genes cysG and oriT as determined by Droplet Digital PCR. The gDNA concentration was highest after 10 min treatment. Grey dots—single measurements, colored dots—mean and standard deviation of four replicates. B PCN calculated as the ratio of pDNA (oriT) concentration and gDNA ( [file 12934_2016_610_MOESM3_ESM.pdf]
